# Clinical Approach in the Management of Paediatric Patients with Familial Hypercholesterolemia: A National Survey Conducted by the LIPIGEN Paediatric Group

**DOI:** 10.3390/nu15153468

**Published:** 2023-08-05

**Authors:** Cristina Pederiva, Marta Gazzotti, Marcello Arca, Maurizio Averna, Giuseppe Banderali, Giacomo Biasucci, Marta Brambilla, Paola Sabrina Buonuomo, Paolo Calabrò, Francesco Cipollone, Nadia Citroni, Sergio D’Addato, Maria Del Ben, Simonetta Genovesi, Ornella Guardamagna, Gabriella Iannuzzo, Lorenzo Iughetti, Giuseppe Mandraffino, Lorenzo Maroni, Giuliana Mombelli, Sandro Muntoni, Fabio Nascimbeni, Angelina Passaro, Fabio Pellegatta, Matteo Pirro, Livia Pisciotta, Roberta Pujia, Riccardo Sarzani, Roberto Scicali, Patrizia Suppressa, Sabina Zambon, Maria Grazia Zenti, Sebastiano Calandra, Alberico Luigi Catapano, Patrizia Tarugi, Federica Galimberti, Manuela Casula, Maria Elena Capra

**Affiliations:** 1 Clinical Service for Dyslipidaemias, Study and Prevention of Atherosclerosis in Childhood, Paediatrics Unit, ASST-Santi Paolo e Carlo, 20142 Milan, Italy; 2 Fondazione SISA (Società Italiana per lo Studio dell’Aterosclerosi), 20133 Milan, Italy; 3 Dipartimento di Medicina Traslazionale e di Precisione, Università La Sapienza di Roma, 00185 Rome, Italy; 4 AO Policlinico Umberto I, 00161 Rome, Italy; 5 Department of Health Promotion, Mother and Child Care, Internal Medicine and Medical Specialties, University of Palermo, 90127 Palermo, Italy; 6 Istituto di Biofisica, Consiglio Nazionale Delle Ricerche, 90146 Palermo, Italy; 7 Centre for Paediatric Dyslipidaemias, Paediatrics and Neonatology Unit, Guglielmo da Saliceto Hospital, 29121 Piacenza, Italy; 8 Unit of Cell and Molecular Biology in Cardiovascular Diseases, Centro Cardiologico Monzino IRCCS, 20138 Milan, Italy; 9 Rare Diseases and Medical Genetic Unit, Ospedale Pediatrico Bambino Gesù, IRCCS, 00163 Rome, Italy; 10 UOC Cardiologia Clinica a Direzione Universitaria e UTIC, AORN “Sant’Anna e San Sebastiano”, 81100 Caserta, Italy; paolo.calabro@unicampania.it; 11 Dipartimento di Scienze Mediche Traslazionali, Università Degli Studi Della Campania “Luigi Vanvitelli”, 80131 Naples, Italy; 12 Clinica Medica, Centro di Alta Specializzazione per la Prevenzione dell’Aterosclerosi, Centro di Eccellenza ESH per L’ipertensione Arteriosa, Centro di Riferimento Regionale per le Dislipidemie, Ospedale Policlinico SS Annunziata, 66100 Chieti, Italy; 13 Centro Dislipidemie e Aterosclerosi, Ospedale di Trento, APSS-Trento, 38122 Trento, Italy; 14 UO di Medicina Interna Cardiovascolare, Ambulatorio Dislipidemie, Università di Bologna, 40138 Bologna, Italy; 15 IRCCS S Orsola, 40138 Bologna, Italy; 16 Dipartimento Scienze Cliniche, Internistiche, Anestesiologiche e Cardiovascolari, Università La Sapenza di Roma, 00161 Rome, Italy; 17 Istituto Auxologico Italiano, 20149 Milan, Italy; 18 Dipartimento di Medicina e Chirurgia, Università di Milano-Bicocca, 20126 Milan, Italy; 19 Department of Public Health and Paediatric Sciences, Turin University, 10126 Turin, Italy; 20 Dipartimento di Medicina Clinica e Chirurgia, Università degli Studi di Napoli Federico II, 80131 Naples, Italy; 21 U.O.C. Pediatria, Azienda Ospedaliero Universitaria di Modena, 41124 Modena, Italy; 22 Department of Clinical and Experimental Medicine, Lipid Centre, University Hospital G Martino, 98100 Messina, Italy; 23 Ambulatorio Ipertensione Dislipidemie, UO Medicina Generale, ASST Valle Olona, Ospedale di Gallarate, 21013 Gallarate, Italy; 24 Centro Dislipidemie ASST Grande Ospedale Metropolitano Niguarda, 20162 Milan, Italy; 25 Dipartimento di Scienze Biomediche, Università Degli Studi di Cagliari, 09124 Cagliari, Italy; 26 Centro per le Malattie Dismetaboliche e l’Arteriosclerosi, Associazione ME DI CO Onlus Cagliari, 09123 Cagliari, Italy; 27 UO Medicina Interna Metabolica, Lipidology Centre, Baggiovara Hospital, AOU of Modena, 41126 Modena, Italy; 28 Department of Biomedical, Metabolic and Neural Sciences, University of Modena and Reggio Emilia, 41125 Modena, Italy; 29 Centro per lo Studio e il Trattamento Delle Malattie del Metabolismo, Aterosclerosi e Nutrizione Clinica, Azienda Ospedaliera-Universitaria S Anna di Ferrara, 44124 Ferrara, Italy; 30 Dipartimento di Medicina Traslazionale e per la Romagna, Università degli Studi di Ferrara, 44122 Ferrara, Italy; 31 IRCCS MultiMedica, 20099 Sesto San Giovanni, Italy; 32 Centro per lo Studio dell’Aterosclerosi, Ospedale E Bassini, 20092 Cinisello Balsamo, Italy; 33 Sezione Medicina Interna, Angiologia e Malattie da Arteriosclerosi, Dipartimento di Medicina e Chirurgia, Università Degli Studi di Perugia, 06132 Perugia, Italy; 34 IRCCS Ospedale Policlinico San Martino UOSD Dietetica e Nutrizione Clinica, Dipartimento di Medicina Interna, Università di Genova, 16132 Genoa, Italy; 35 Dipartimento Scienze Mediche Chirurgiche, Università Degli Studi Magna Graecia, 88100 Catanzaro, Italy; 36 Clinica Medica e Geriatrica, Dipartimento di Scienze Cliniche e Molecolari, Università Politecnica Delle Marche, 60126 Ancona, Italy; 37 IRCCS-INRCA, 60124 Ancona, Italy; 38 Department of Clinical and Experimental Medicine, University of Catania, Ospedale Garibaldi, 95122 Catania, Italy; 39 Department of Internal Medicine and Rare Diseases Centre “C. Frugoni”, University Hospital of Bari, 70124 Bari, Italy; patrizia.suppressa@gmail.com; 40 Dipartimento di Medicina, Università di Padova, 35128 Padua, Italy; 41 Servizio di Diabetologia e Malattie Metaboliche “Ospedale P. Pederzoli”, Casa di Cura Privata, 37019 Peschiera del Garda, Italy; 42 Department of Life Sciences, University of Modena and Reggio Emilia, 41125 Modena, Italy; 43 Epidemiology and Preventive Pharmacology Service (SEFAP), Department of Pharmacological and Biomolecular Sciences, University of Milan, 20133 Milan, Italy

**Keywords:** familial hypercholesterolemia, paediatric, pathology register, survey, management

## Abstract

Detection and treatment of patients with familial hypercholesterolemia (FH) starting from childhood is fundamental to reduce morbidity and mortality. The activity of National realities such as the LIPIGEN (LIpid transPort disorders Italian GEnetic Network) Paediatric Group, founded in 2018, is a milestone in this context. The aim of this exploratory survey, conducted in October 2021 among Italian lipid clinics included in the LIPIGEN Paediatric Group, was to investigate the current clinical approach in the management and treatment of paediatric patients with suspected FH. A digital questionnaire composed of 20 questions investigating nutritional treatment and nutraceutical and pharmacological therapy for children and adolescents with FH was proposed to the principal investigators of 30 LIPIGEN centres. Twenty-four centres responded to the section referring to children aged < 10 years and 30 to that referring to adolescents. Overall, 66.7% of children and 73.3% of adolescents were given lipid-lowering nutritional treatment as the first intervention level for at least 3–4 months (29.2% and 23.3%) or 6–12 months (58.3% and 53.3%). Nutraceuticals were considered in 41.7% (regarding children) and 50.0% (regarding adolescents) of the centres as a supplementary approach to diet. Lipid-lowering drug therapy initiation was mainly recommended (91.7% and 80.0%). In 83.3% of children and 96.7% of adolescents, statins were the most frequently prescribed drug. We highlighted several differences in the treatment of paediatric patients with suspected FH among Italian centres; however, the overall approach is in line with the European Atherosclerosis Society (EAS) recommendations for FH children and adolescents. We consider this survey as a starting point to reinforce collaboration between LIPIGEN centres and to elaborate in the near future a consensus document on the management of paediatric patients with suspected FH so as to improve and uniform detection, management, and treatment of these patients in our country.

## 1. Introduction

Heterozygous familial hypercholesterolemia (FH) is a very common inherited disease, as it involves approximately 1 subject in 250 in the general population [1,2]. Subjects with FH have altered blood lipid profile with elevated total cholesterol (TC) and low-density lipoprotein cholesterol (LDL-C), thus leading to an accelerated atherosclerotic process [3]. Detection and treatment of subjects with FH starting from childhood is an issue of utmost importance, and it helps the patient “gain decades of life”, as stated in the International Consensus Statement [1]. FH is still underdiagnosed and undertreated in the general population [3]. Diagnosis and treatment of paediatric subjects with FH is even more challenging both because the knowledge of disease among paediatricians is still low, and its management must be different with respect to adult subjects. Current consensus documents [1] on management of paediatric patients with hypercholesterolaemia agree that nutritional intervention is the first-line treatment in this category of patients. The Mediterranean diet is an optimal dietary pattern with a balanced intake of macronutrients and micronutrients, and it is widely adopted in our country. The Mediterranean diet implies a high consumption of fruits, vegetables, whole cereals, low-fat meat, poultry, pulses, and fish, with daily consumption of olive oil. Nutraceuticals with lipid-lowering effects can be used from 6 years of age, and they can be added to nutritional interventions so as to enhance the lipid-lowering effect and to reach lower plasma LDL-C levels. Pharmacological treatment is feasible from 8 years of age with pravastatin and from 10 years of age with other statins with higher lipid-lowering action. Pharmacological treatment must be adjusted according to the patient’s plasma LDL-C levels, and these must be monitored with scheduled control visits. The current guidelines [1] also indicate how to start therapy in paediatric patients with FH, but clinicians should remember that children and adolescents cannot be considered as “little adults”, and they and their families need a more tailored and personalized approach both for nutritional and the pharmacological treatment [4].

In order to verify possible limitations to the application of guidelines and to further the collection of information that can support shared recommendations, it is essential to know the approach used in current clinical practice. The implementation of a National Pathology Register has been very useful in this context, as it allowed to focus on the detection and treatment of subjects with FH and also on specific subgroups, such as subjects aged less than 18 years [5,6]. In Italy, the LIPIGEN (LIpid transPort disorders Italian GEnetic Network) study is an observational, multicentre study sponsored by the Foundation of Italian Atherosclerosis Society (SISA) and established since 2009 to promote the diagnosis of genetic dyslipidaemias, with a primary focus on FH [7]. The LIPIGEN Network is constituted by lipid centres specialized in the diagnosis, management, and treatment of genetically determined dyslipidaemias. Medical doctors working in LIPIGEN centres have different and various residencies, such as internal medicine, cardiology, and paediatrics, while they all have nutritional expertise and are educated on new and updated lipid-lowering therapies. In some centres, dieticians are part of the LIPIGEN staff as well. In 2018, a subgroup of Italian Lipid Clinics involved in the LIPIGEN Network constituted the LIPIGEN Paediatric Group [8]. This sub-study involves both centres specifically dedicated to paediatric patients and adult centres dealing with paediatric patients as well. The aim of the LIPIGEN Paediatric Group is to improve the diagnosis, detection, and treatment of children and adolescents with FH [5]. In October 2021, the LIPIGEN Paediatric Group accounted for over than 1600 subjects aged less than 18 years with a clinical and/or genetic diagnosis of FH who were followed-up by the 30 LIPIGEN centres widely distributed in the whole Italian national territory, with 5 of them specifically dedicated to the paediatric population [8].

The aim of this exploratory survey is to investigate the current clinical approach in the management and treatment of children and/or adolescents with suspected FH, providing the basis to improve the data collection for paediatric follow-up analysis.

## 2. Methods

This survey was conducted among Italian lipid clinics included in the LIPIGEN Paediatric group. In October 2021, a digital link was provided to the principal investigators of 30 LIPIGEN centres, which include specialized paediatric clinics or adult lipid clinics that also manage individuals younger than 18 years of age with a suspected FH diagnosis.

The questionnaire is composed by a total of 20 questions in the Italian language (11 multiple choice and 9 open-ended questions), repeated in two identical sections referring to (i) children < 10 years and (ii) adolescents ≥ 10 years (Supplementary Table S1). Questions are repeated for these two different age groups so as to have a more precise and accurate description of the management and treatment of paediatric patients with suspected FH: younger patients are mainly treated with nutritional intervention, and sometimes nutraceuticals are added to this first-line treatment, whereas adolescents often receive pharmacological therapy as well starting from the age of 10 years. The principal investigators were required to choose the answers that were a better fit with their normal clinical practice.

The main topics of the survey refer to the following:

- Diet: type of diet, professional figure in charge of its preparation, how long it is maintained, and how useful it is considered;
- Nutraceutical approach: when it is recommended, the patient’s compliance and the parents’ concordance with the use of nutraceuticals, and how useful it is considered;
- Pharmacological therapy: when it is considered/started, with which drug it is generally started, and patient’s compliance and parents’ concordance with the drug use.

## 3. Statistical Analysis

Survey responses were imported into IBM SPSS Statistics for Windows, version 28 (IBM Corp., Armonk, NY, USA). The prevalence of reported answers is described as percentages.

## 4. Results

Out of 30 centres to which the survey was sent, 24 responded to the part referring to children aged < 10 years and 30 to the part referring to adolescents aged ≥ 10 years.

In almost all centres, after a suspected diagnosis of FH, both children and adolescents receive lifestyle and dietary–nutritional indications, mainly consisting of a low-fat diet with qualitative indications (66.7% of children and 73.3% of adolescents), sometimes custom-developed by a dietician (25.0% and 26.7%, respectively), for at least 3–4 months (29.2% and 23.3%) or 6–12 months (58.3% and 53.3%, respectively) before starting a pharmacological treatment. The main results on nutritional intervention are summarized in Table 1. Compliance to dietary–nutritional indications was reported to be good (at least 8 on a 1–10 scale) in 43.7% (for children) and 52.4% (for adolescents) of the centres.

**Figure 1:**
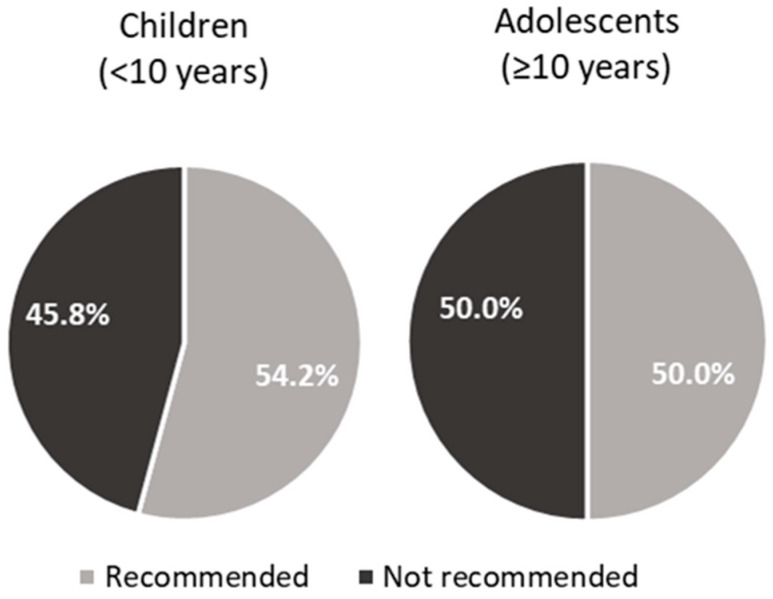
Recommendations for the use of nutraceuticals/food supplements in children (<10 years) and adolescents (≥10 years).

The use of nutraceuticals or food supplements was documented in 54.2% of children (in 3 out of 5 (60%) centres specifically dedicated to the paediatric population) and in 50.0% of subjects ≥ 10 years (Figure 1).

In 41.7% (for children) and 50.0% (for adolescents) of the centres, the use of nutraceuticals or food supplements is considered as a supplementary approach to diet before moving on to pharmacological intervention. When administrated, this approach includes the prescription of phytosterols (eight centres reporting the use in children and nine centres in adolescents), monacolin K (six centres reporting the use in children and eight centres in adolescents), and seldom berberine, glucomannan, and polyunsaturated fatty acids, usually without the onset of any adverse events. (Only myalgia associated with monacolin K was reported.) Moreover, on a 1–10 scale, the mean compliance among patients with nutraceuticals/food supplements was reported as quite good and almost comparable between young patients and adolescents (6.9 vs. 7.2), with a 43.8% (referring to children) and 52.4% (referring to adolescents) reporting values of 8 or more. However, three centres reported a compliance rate lower than 5 for adolescents, while only one centre reported a compliance lower than 5 for children to nutraceuticals/food supplements. The mean concordance of parents for nutraceuticals/food supplements intake was also good and comparable between the two age groups (7.9 vs. 8.1).

**Figure 2:**
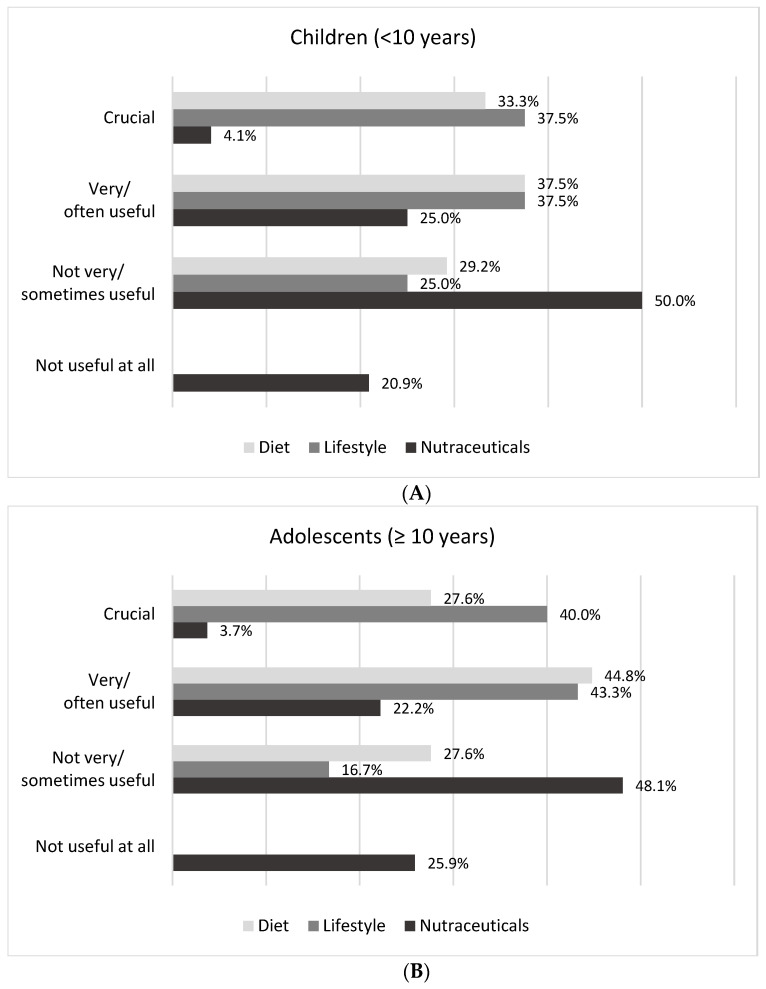
Evaluation of the usefulness of dietary–nutritional approach (diet), lifestyle modification (lifestyle), and nutraceutical intervention (nutraceuticals) in children (<10 years) (**A**) and in adolescents (≥10 years) (**B**) by Italian lipid clinics.

Analysing the therapeutic usefulness of the dietary–nutritional approach, lifestyle modification, and = nutraceutical intervention, the implementation of dietary and lifestyle habits in children and subjects ≥ 10 years was mainly reported to be crucial/very often useful (70.8% and 75.0% in children vs. 72.4% and 83.3% in adolescents), while the use of nutraceuticals was not perceived as useful in 70.9% and 74.0% of cases, respectively (Figure 2).

The analysis of the answers relating to the pharmacological treatment showed that lipid-lowering therapy initiation is mainly recommended regardless of the age (91.7% of subjects with LDL-C ≥ 190 mg/dL and 80.0% of subjects with LDL-C ≥ 160 mg/dL, plus other risk factors).

The lipid-lowering drugs more frequently prescribed as a first step are statins (83.3% in children and 96.7% in adolescents), mainly including atorvastatin, pravastatin, and rosuvastatin, with lower dosages at younger ages, and/or ezetimibe (29.2% and 30.0%, respectively). Cholestyramine is rarely prescribed (N = 3 and N = 2, respectively) (Table 2).

Responders documented a rather low rate of side effects to the drugs as reported by patients: mild myalgias, asthenia, and muscle toxicity in association with statin therapy and cases of gastrointestinal adverse effects associated with cholestyramine treatment, with the only report of an increased frequency of myalgias in statin-treated adolescents who practice intense sporting activity.

The mean patient compliance to pharmacological therapy on a scale from 1 to 10 did not differ between children and adolescents (8.1 in both groups), and the concordance of parents with the administration of lipid-lowering treatment was the same among children and subjects ≥ 10 years.

## 5. Discussion

This is the first national survey conducted in Italy investigating the clinical management and therapeutic approach of paediatric patients with suspected FH among lipid clinics. The survey mainly focused on three topics: nutritional intervention, nutraceutical use, and pharmacological therapy.

### 5.1. Nutritional Intervention

Nutritional treatment and lifestyle modification were the first-step interventions in most of the involved lipid clinics; nutritional treatment lasted at least three months before starting drug therapy. Most of the operators perceived nutritional treatment as useful or very useful, and the reported compliance to nutritional intervention was shown to be sub-optimal. Nutritional intervention is mainly dealt with by the medical doctor, and only in 25% of cases it is carried out by a dietician. Nutritional intervention is performed for at least 6–12 months in most centres before adding another therapy (58.3% for children and 53% for adolescents), which is in line with the European Atherosclerosis Society (EAS) recommendations, so as to reach a heart-healthy diet [9]. This finding is not unexpected since nutritional intervention in young patients with FH is not a strict diet but a tailored nutritional counselling aimed at achieving a healthy-heart lifestyle; in addition, most of the lipid centres deal with adult subjects, and staff dedicated only to dietetic intervention are not always present. We are currently working on this aspect so as to elaborate nutritional indications that are more specific and targeted for paediatric patients with FH. According to data available so far, nutritional lipid-lowering treatment is safe [10,11,12,13], and if well established, it remains a lifestyle model for the patient to follow for their lifetime.

### 5.2. Nutraceutical Use

We included nutraceuticals in our survey, as they are nutritional compounds increasingly used and studied in patients with FH during the last decades [9,14,15]. The use of nutraceuticals or food supplements is recommended in 54.2% of children and 50.0% of subjects ≥ 10 years by the interviewed centres. Nutraceuticals are considered as a supplementary therapy in patients who are on nutritional treatment by almost half of the centres. The most widely used products are phytosterols and monacolin K. Phytosterols are included in International Guidelines for the treatment of paediatric patients with FH [16], and they have a proven lipid-lowering activity and a good safety profile [17,18]. The survey was conducted in October 2021; therefore, monacolin K is still mentioned among useable nutraceuticals for paediatric patients with suspected FH. In June 2022, the European Commission Regulation stated that the individual portion of a nutraceutical for daily consumption must provide less than 3 mg of monacolins from red yeast rice. Therefore, all food supplements with a daily dose of monacolins from red yeast rice greater than or equal to 3 mg/day will be banned at the European level, and mandatory mentions and warnings will be required on the labels of food supplements with a daily dose of monacolins from red yeast rice. Furthermore, monacolin K cannot be used anymore for patients aged less than 18 years [19]. Fibres are not used in most centres even if they can be considered as a food supplement for paediatric patients with FH and if their lipid-lowering effect has been recognized by the European Food Safety Authority (EFSA) [20]. Nutraceuticals containing fibres and phytosterols should always be considered as a complement to dietary and lifestyle interventions. However, since there are no long-term safety trials and no large intervention trials, these nutritional compounds should be used for a short period as a complement to nutritional intervention or in subjects who cannot use drug therapy yet due to their young age or to their mild hypercholesterolemia [15,21].

### 5.3. Pharmacological Therapy

Pharmacological lipid-lowering therapy is initiated in case of severe hypercholesterolemia (LDL-C ≥ 190 mg/dL or LDL-C ≥ 160 mg/dL, with additional risk factors) in the majority of centres (91.7% and 80.0%, respectively). Statins are the most frequently prescribed drugs. This is in line with the current international recommendations [1]. There is no concordance on the specific statin to use as a first-line therapy: pravastatin is one the most studied, but it has an inferior lipid-lowering effect if compared to other molecules that, on the contrary, are more powerful but have been studied for fewer years and might have more side effects [1,22,23,24]. Nowadays, all the main consensus documents [1,9,16] agree on statin use in paediatric patients with FH. However, this topic is still widely debated both for the specific statin to start with and for the appropriate dosage. We found that the treatments used were not uniformly distributed among centres. This is a quite expected finding, but it represents a barrier to the analysis of follow-up data and to the evaluation of the data quality. This is another point we are planning to develop so as to create a consensus document to be used in all centres.

### 5.4. Strengths and Limitations

The LIPIGEN Paediatric Network includes 30 lipid centres located in almost all Italian regions, dealing with more than 1600 patients aged less than 18 years, thus configuring itself as one of the largest paediatric FH cohorts in Europe. Another strength of our survey is that we evaluated real-life data, thus providing an updated and realistic picture of the management of paediatric patients with suspected FH throughout the Italian territory.

Our survey is not free from limitations. First, the numerical imbalance between “mixed” centres (dealing with both adult and paediatric patients) and paediatric centres (dealing only with children and adolescents) did not allow us to carry out stratified analyses. Second, in some “mixed” centres, the experience and know-how on subjects aged less than 18 years could be limited. Nevertheless, the presence of LIPIGEN centres specifically dedicated to the detection and management of paediatric patients with suspected FH and the presence of a strong and well-established national network is without a doubt a strength with respect to other countries where there are few centres or only ones often geographically distant, with no ability to contribute to a factual and effective cooperation and collaboration. Last, data were collected by the doctor/researcher; therefore, the survey does not reflect the patients’ point of view and may give an incomplete picture of the actual acceptability and sustainability of nutritional and pharmacological approaches.

## 6. Conclusions

In our survey, we aimed to provide a real-life picture of the approach of LIPIGEN Paediatric centres in the management of patients aged less than 18 years with suspected FH. We analysed the main treatments used for FH in children and adolescents. We highlighted that there is no single, standardized approach in all centres and that some points still need be addressed. However, the intervention strategies are in line with those recommended in the International Consensus Documents. Our survey carried out throughout the LIPIGEN Paediatric Network should be considered as the starting point to improve the detection, management, treatment, and outcome of children and adolescents with a clinical diagnosis of FH in Italy.

## Figures and Tables

**Table 1:** Nutritional intervention strategies according to age groups.

Nutritional Intervention	Children	Adolescents
Qualitative recommendations given by medical doctors	66.7%	73.3%
Qualitative recommendations given by dietician	25.0%	26.7%
Lasting for 3–4 months	29.2%	23.3%
Lasting for 6–12 months	58.3%	53.3%

**Table 2:** List of drugs usually prescribed to start the pharmacological therapy in children (<10 years) and adolescents (≥10 years), ordered by frequency of active pharmaceutical ingredient prescription.

Children (<10 Years)	Adolescents (≥10 Years)
Pravastatin 10–20–40 mg (N = 13)	Rosuvastatin 5–10 mg (N = 18)
Rosuvastatin 2.5–5–10 mg (N = 10)	Pravastatin 10–20–40 mg (N = 14)
Atorvastatin 10 mg (N = 7)	Atorvastatin 10 mg (N = 14)
Ezetimibe (N = 7)	Ezetimibe (N = 9)
Cholestyramine (N = 3)	Cholestyramine (N = 2)

## Data Availability

The data that support the findings of this study are available from the corresponding author, M.C., upon reasonable request.

## Supplementary Materials

The following supporting information can be downloaded at: https://www.mdpi.com/article/10.3390/nu15153468/s1, Supplementary Table S1: Questionnaire (English translation).

## Abbreviations

EFSA	European Food Safety Authority
FH	Familial hypercholesterolemia
LIPIGEN	LIpid transPort disorders Italian GEnetic Network
LDL-C	Low-density lipoprotein cholesterol
TC	Total cholesterol

## References

[1] Wiegman A., Gidding S.S., Watts G.F., Chapman M.J., Ginsberg H.N., Cuchel M., Ose L., Averna M., Boileau C., Borén J. (2015). Familial Hypercholesterolaemia in Children and Adolescents: Gaining Decades of Life by Optimizing Detection and Treatment. Eur. Heart J..

[2] Akioyamen L.E., Genest J., Shan S.D., Reel R.L., Albaum J.M., Chu A., Tu J.V. (2017). Estimating the Prevalence of Heterozygous Familial Hypercholesterolaemia: A Systematic Review and Meta-Analysis. BMJ Open.

[3] Nordestgaard B.G., Chapman M.J., Humphries S.E., Ginsberg H.N., Masana L., Descamps O.S., Wiklund O., Hegele R.A., Raal F.J., Defesche J.C. (2013). FamilialHypercholesterolaemia Is Underdiagnosed and Undertreated in the General Population:Guidance for Clinicians to Prevent Coronary Heart Disease: Consensus Statement of the European Atherosclerosis Society. Eur. Heart J..

[4] Pederiva C., Capra M., Viggiano C., Rovelli V., Banderali G., Biasucci G. (2021). Early Prevention of Atherosclerosis: Detection and Management of Hypercholesterolaemia in Children and Adolescents. Life.

[5] Gazzotti M., Casula M., Olmastroni E., Averna M., Arca M., Catapano A.L. (2020). How registers could enhance knowledge and characterization of genetic dyslipidaemias: The experience of the LIPIGEN in Italy and of other networks for familial hypercholesterolemia. Atheroscler. Suppl..

[6] de Ferranti S.D., Shrader P., Linton M.F., Knowles J.W., Hudgins L.C., Benuck I., Kindt I., O’Brien E.C., Peterson A.L., Ahmad Z.S. (2021). Children with Heterozygous Familial Hypercholesterolemia in the United States: Data from the Cascade Screening for Awareness and Detection-FH Registry. J. Pediatr..

[7] Averna M., Cefalù A.B., Casula M., Noto D., Arca M., Bertolini S., Calandra S., Catapano A.L., Tarugi P., LIPIGEN Group (2017). Familial Hypercholesterolemia: The Italian Atherosclerosis Society Network (LIPIGEN). Atheroscler. Suppl..

[8] Gazzotti M., Casula M., Bertolini S., Capra M.E., Olmastroni E., Catapano A.L., Pederiva C. (2022). The Role of Registers in Increasing Knowledge and Improving Management of Children and Adolescents Affected by Familial Hypercholesterolemia: The LIPIGEN Pediatric Group. Front. Genet..

[9] Mach F., Baigent C., Catapano A.L., Koskinas K.C., Casula M., Badimon L., Chapman M.J., De Backer G.G., Delgado V., Ference B.A. (2020). 2019 ESC/EAS Guidelines for the management of dyslipidaemias: Lipid modification to reduce cardiovascular risk. Eur. Heart J..

[10] Obarzanek E., Kimm S.Y.S., Barton B.A., Van Horn L., Kwiterovich P.O., Simons-Morton D.G., Hunsberger S.A., Lasser N.L., Robson A.M., Franklin F.A. (2001). Long-Term Safety and Efficacy of a Cholesterol-Lowering Diet in Children With Ele-vated Low-Density Lipoprotein Cholesterol: Seven-Year Results of the Dietary Intervention Study in Children (DISC). Pediatrics.

[11] Rask-Nissilä L., Jokinen E., Terho P., Tammi A., Hakanen M., Rönnemaa T., Viikari J., Seppänen R., Välimäki I., Helenius H. (2002). Effects of diet on the neurologic development of children at 5 years of age: The STRIP project. J. Pediatr..

[12] Capra M.E., Pederiva C., Viggiano C., Fabrizi E., Banderali G., Biasucci G. (2022). Nutritional Treatment in a Cohort of Pediatric Patients with Familial Hypercholesterolaemia: Effect on Lipid Profile. Nutrients.

[13] Rodríguez-Borjabad C., Narveud I., Christensen J.J., Ulven S.M., Malo A.I., Ibarretxe D., Girona J., Torvik K., Bogsrud M.P., Retterstøl K. (2021). Dietary intake and lipid levels in Norwegian and Spanish children with familial hypercholesterolemia. Nutr Metab. Cardiovasc. Dis..

[14] Gylling H., Plat J., Turley S., Ginsberg H.N., Ellegård L., Jessup W., Jones P.J., Lütjohann D., Maerz W., Chapman M.J. (2014). Plant sterols and plant stanols in the management of dyslipidaemia and prevention of cardiovascular disease. Atherosclerosis.

[15] Casula M., Catapano A.L., Magni P. (2022). Nutraceuticals for Dyslipidaemia and Glucometabolic Diseases: What the Guidelines Tell Us (and Do Not Tell, Yet). Nutrients.

[16] (2011). Expert Panel on Integrated Guidelines for Cardiovascular Health and Risk Reduction in Children and Adolescents; National Heart, Lung, and Blood Institute. Expert panel on integrated guidelines for cardiovascular health and risk reduction in children and adolescents: Summary report. Pediatrics.

[17] Andersson S.W., Skinner J., Ellegård L., Welch A.A., Bingham S., Mulligan A., Andersson H., Khaw K.T. (2004). Intake of dietary plant sterols is inversely related to serum cholesterol concentration in men and women in the EPIC Norfolk population: A cross-sectional study. Eur. J. Clin. Nutr..

[18] Klingberg S., Ellegård L., Johansson I., Hallmans G., Weinehall L., Andersson H., Winkvist A. (2008). Inverse relation between dietary intake of naturally occurring plant sterols and serum cholesterol in northern Sweden. Am. J. Clin. Nutr..

[19] COMMISSION REGULATION (EU) 2022/860 of 1 June 2022 amending Annex III to Regulation (EC) No 1925/2006 of the European Parliament and of the Council as regards monacolins from red yeast rice. Official Journal of the European Union. L 151/37. June 2022. https://eur-lex.europa.eu/eli/reg/2022/860/oj.

[20] (2010). EFSA Panel on Dietetic Products, Nutrition, and Allergies (NDA), European Food Safety Authority Scientific opinion on dietary reference values for carbohydrates and dietary fiber. EFSA J..

[21] Banderali G., Capra M.E., Viggiano C., Biasucci G., Pederiva C. (2022). Nutraceuticals in paediatric patients with dyslipidaemia. Nutrients.

[22] Pang J., Chan D.C., Watts G.F. (2020). The Knowns and Unknowns of Contemporary Statin Therapy for Familial Hypercholesterolemia. Curr. Atheroscler. Rep..

[23] Mamann N., Lemale J., Karsenty A., Dubern B., Girardet J.P., Tounian P. (2019). Intermediate-term efficacy and tolerance of statins in children. J. Pediatr..

[24] Vuorio A., Kuoppala J., Kovanen P.T., E Humphries S., Tonstad S., Wiegman A., Drogari E., Ramaswami U. (2019). Statins for children with familial hypercholesterolemia. Cochrane Database Syst..

