# Supplementary material for: Clinical Approach in the Management of Paediatric Patients with Familial Hypercholesterolemia: A National Survey Conducted by the LIPIGEN Paediatric Group
Source: Nutrients. 2023 Aug 5;15(15):3468. doi: 10.3390/nu15153468 (PMC10420921; doi:10.3390/nu15153468)
Supplement: Supplementary file 1 [file nutrients-15-03468-s001.zip › nutrients-2500479-supplementary.pdf]

nutrients

Article

Clinical Approach in the Management of Paediatric Patients with Familial Hypercholesterolemia: A National Survey Conducted by the LIPIGEN Paediatric Group

Supplementary Table S1 - Questionnaire (English translation)

1	Has the child/adolescent received any indications before starting drug therapy?<br>• No<br>• Yes, dietary-nutritional indications<br>• Yes, lifestyle change indications<br>• Yes, both dietary-nutritional and lifestyle change indications
2	If yes, for how long is the child/adolescent's diet maintained before starting nutraceuticals or drug therapy?<br>• 4-8 weeks<br>• 3-4 months<br>• 6-8 months<br>• 12 months
3	What diet intervention is usually recommended for the child/adolescent?<br>• Very low-fat diet (lipids ≤ 25%)<br>• Moderate low-fat diet (lipids ≤ 30%)<br>• Low-fat diet (lipids 25-30%) with portions<br>• Low-fat diet (lipids 25-30%) with qualitative advices
4	The diet for the child/adolescent:<br>• Is developed by the dietician<br>• Is developed by the nutritionist physician<br>• Consists of a pre-printed pattern<br>• Consists of qualitative advice derived from food recall or other survey method
5	The compliance with the dietary intervention proposed to the child/adolescent is generally:<br>• Poor<br>• Good<br>• Excellent<br>• Dependent on the frequency of counselling
6	Was the child/adolescent recommended to take nutraceuticals or food supplements?<br>• No<br>• Yes, before the pharmacological therapy<br>• Yes, as an alternative to pharmacological therapy<br>• Yes, in combination to start of the pharmacological therapy
7	Which nutraceutical-food supplement is mainly indicated as initial approach in child/adolescent?
8	What is the child/adolescent's compliance with the nutraceutical-food supplement on a scale from 1 to 10?

9	What is the concordance of the child/adolescent's parents with assumption of the nutraceutical-food supplement on a scale from 1 to 10?
10	What are the main adverse effects developed by the child/adolescent during the use of nutraceuticals-food supplements?
11	How do you evaluate the usefulness of the dietary/nutritional approach in the management of FH in the child/adolescent, according to your experience?<br>• Not useful at all<br>• Not very/sometimes useful<br>• Very/often useful<br>• Crucial
12	How do you evaluate the usefulness of lifestyle modifications in the management of FH in the child/adolescent, according to your experience?<br>• Not useful at all<br>• Not very/sometimes useful<br>• Very/often useful<br>• Crucial
13	How do you evaluate the usefulness of nutraceutical interventions in the management of FH in the child/adolescent, according to your experience?<br>• Not useful at all<br>• Not very/sometimes useful<br>• Very/often useful<br>• Crucial
14	Do you consider the pharmacological therapy in child/adolescent?<br>• No<br>• Yes, only in severe cases (LDL-C ≥ 190 mg/dL or LDL-C ≥ 160 mg/dL + risk factors)<br>• Yes, only if the child/adolescent does not respond/respect the diet<br>• Yes, only if the child/adolescent does not respond to the nutraceutical supplement
15	In child/adolescent, the pharmacological therapy is usually started with<br>• Statin<br>• Other drug
16	If statin, which statin(s) and at what dosage?
17	If other drug, which drug and at what dosage?
18	What is the child/adolescent's compliance with the pharmacological therapy on a scale from 1 to 10?
19	What is the concordance of the child/adolescent's parents with pharmacological therapy on a scale from 1 to 10?
20	What are the main adverse effects developed by the child/adolescent during the pharmacological therapy?
